# Comment on ‘Overall Mortality for Community‐Dwelling Adults Over 50 Years at Risk of Malnutrition’ by Gittins et al.

**DOI:** 10.1002/jcsm.13632

**Published:** 2024-11-06

**Authors:** Yizhuan Huang, Han Wang

**Affiliations:** ^1^ Affiliated Sport Hospital of Chengdu Sport University Chengdu China

To the Editor:

We are writing in response to the article titled ‘Overall Mortality for Community‐Dwelling Adults Over 50 Years at Risk of Malnutrition’ [1]. This study highlights the significant association between malnutrition risk and overall mortality, underscoring the importance of broader nutritional screening at the community level. We commend the authors for their valuable contribution and would like to offer a few suggestions for consideration.

First, the study's sample is primarily drawn from the UK, lacking direct comparisons with other countries or regions. As a result, the findings may not be generalizable to older adults in different cultural contexts or healthcare systems, pointing to a clear geographical limitation.

Second, while the study performed subgroup analyses on cancer patients and gender, future research could benefit from further stratification by age groups, socioeconomic status, dietary habits and psychological health [2, 3, 4]. Such analyses would provide more precise insights and help tailor more targeted and personalized intervention strategies.

Third, the study relies not only on weight change but also on other self‐reported data, which may be subject to recall bias or social desirability bias, potentially skewing the results. Future research could enhance accuracy by incorporating objective measures such as blood pressure, blood glucose levels, cholesterol levels and body fat percentage, thereby reducing potential errors.

This study effectively raises awareness of the prevalence and significant impact of malnutrition on the health of middle‐aged and older adults. As healthcare professionals, we advocate for the early identification of malnutrition risk through enhanced nutritional screening and assessment. By collaborating across disciplines with dietitians, physicians, psychologists and social workers, we can develop and implement comprehensive nutritional management plans [5].

In conclusion, we greatly appreciate the insights this study provides into the relationship between malnutrition risk and mortality. Building on this research, we can formulate more targeted nutritional management strategies that improve individual health, extend life expectancy and reduce the burden on healthcare systems.

## Conflicts of Interest

The authors declare no conflicts of interest.
